# Solid-State Synthesis of Polyaniline/Single-Walled Carbon Nanotubes: A Comparative Study with Polyaniline/Multi-Walled Carbon Nanotubes

**DOI:** 10.3390/ma5071219

**Published:** 2012-07-16

**Authors:** Tursun Abdiryim, Aminam Ubul, Ruxangul Jamal, Adalet Rahman

**Affiliations:** Key Laboratory of Petroleum and Gas Fine Chemicals, Educational Ministry of China, College of Chemistry and Chemical Engineering, Xinjiang University, Urumqi 830046, China; E-Mails: aminam520@gmail.com (A.U.); jruxangul@xju.edu.cn (R.J.); adalet@sohu.com (A.R.)

**Keywords:** polyaniline, single-walled carbon nanotubes, composites solid-state polymerization

## Abstract

The polyaniline/single-walled carbon nanotubes (PANI/SWNTs) composites with a content of SWNTs varying from 8 wt% to 32 wt% were synthesized using a solid-state synthesis method. The structure and morphology of the samples were characterized by fourier transform infrared (FTIR) spectra, ultraviolet-visible (UV-vis) absorption spectra, X-ray diffraction (XRD) and transmission electron microscopy (TEM). The electrochemical performances of the composites were investigated by galvanostatic charge–discharge and cycling stability measurements. The structure and properties of PANI/SWNTs were compared with those of PANI/multi-walled carbon nanotubes (PANI/MWNTs) prepared under the same polymerization conditions. The results from FTIR and UV-vis spectra showed that the composites with SWNTs displayed a higher oxidation and doping degree than pure PANI, which is similar to that of PANI/MWNTs. The morphological studies revealed that PANI/SWNTs did not display any rod-like and granular-like features, which appeared in PANI/MWNTs. The galvanostatic charge–discharge measurements indicated that the specific capacitance of PANI/SWNTs is not higher than that of PANI/MWNTs, but the PANI/SWNTs exhibited higher cycling stability and more stable electrochemical behavior in neutral and alkaline electrolytes than PANI/MWNTs.

## 1. Introduction

Supercapacitors, which possess high power density, high cycle efficiency, fast charge/discharge ability and a long cycle life, have attracted considerable attention over the past decades owing to their wide range of potential applications [1]. To develop an advanced supercapacitor device, an active electrode material with high capacity performance is indispensable [2,3]. Carbon materials and their composites are widely used for supercapacitor applications because of their unique properties [4]. Recently, many composites consisting of polyaniline (PANI) with single-walled carbon nanotubes (SWNTs) have been investigated, because of their good flexibility as well as good electrochemical behavior. The PANI/SWNTs composites are widely applied as supercapacitor materials mainly due to the π-π interactions between SWNTs and PANI, which often lead to synergistic effects in improving electrochemical performances of supercapacitors [5]. At the present time, many reports have been published on the preparation of PANI/SWNTs composites [6,7,8]. 

Recently, we have demonstrated a novel room-temperature solid-state oxidative method for the fabrication of PANI/single-walled carbon nanotube (PANI/MWNTs) composites, and we have found that the solid-state polymerization was an effective method for fabricating the composites of carbon nanotubes with a polyaniline type conducting polymer [9]. As an extension of the traditional synthesis method, the solid-state synthesis method has many advantages: reduced pollution, low costs, and simplicity in process and handling. Now, it is widely used for synthesizing polyaniline type conducting polymers [10,11,12].

Herein we report the fabrication of PANI/SWNTs composites as electrode materials for supercapacitors by using solid-state synthesis method. And the correlation between the structures and properties of the PANI/SWNTs composites are discussed based on the results from fourier transform infrared (FTIR), ultraviolet-visible (UV-Vis), X-ray diffraction (XRD) and transmission electron microscopy (TEM) measurements. Moreover, the structure and properties of PANI/SWNTs are compared with those of PANI/MWNTs prepared under the same solid-state polymerization conditions. The effects of the type of carbon nanotubes (single-walled and multi-walled) on the structural and physicochemical properties of the resulting polymers are discussed in detail by comparative studies of FTIR, UV-vis-NIR, X-ray diffraction, TEM, galvanostatic charge–discharge and cycling stability measurements. 

## 2. Results and Discussion

### 2.1. FTIR Spectra

**Figure 1:**
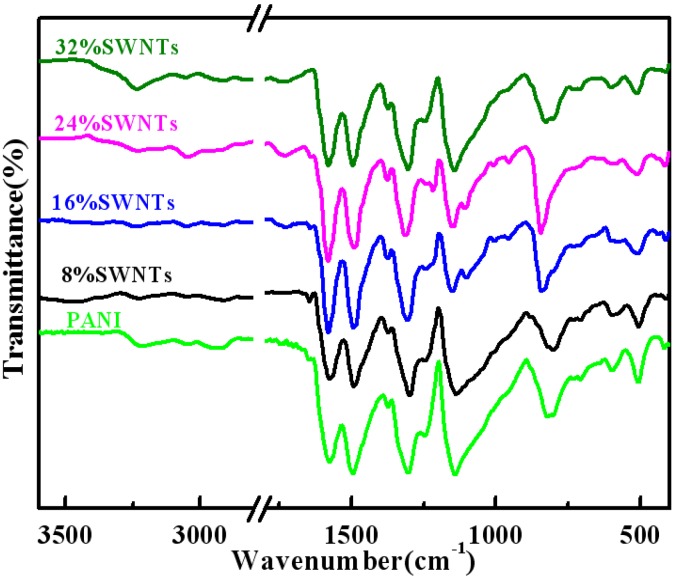
Fourier transform infrared (FTIR) spectra of polyaniline (PANI) and polyaniline/single-walled carbon nanotubes (PANI/SWNTs) composites with a content of SWNTs varying from 8 wt% to 32 wt%.

Figure 1 represents the FTIR spectra of PANI/SWNTs composites and PANI prepared by a solid-state synthesis method. As can be seen in Figure 1, the FTIR spectra of composites are identical to those of PANI. The main characteristic bands of PANI are at 3217 cm^−1^, 2939 cm^−1^, 1577 cm^−1^ and 1496 cm^−1^, 1309 cm^−1^, ~1134 cm^−1^, 820 cm^−1^, and the assignment of these bands are as previously reported [13,14,15]. Compared to PANI, the composites have a higher intensity ratio of quinoid to benzenoid ring modes (~1577 cm^−1^/~1496 cm^−1^), which is similar to the PANI/MWNTs prepared under the same solid-state polymerization conditions [9]. A comparison of the relative intensity ratio of quinoid to benzenoid ring modes in these composites shows that the composite with 24 wt% SWNTs has the highest intensity ratio. However, in our previous report the composite with 16 wt% MWNTs displays the highest intensity ratio in the case of PANI/MWNTs. 

### 2.2. UV-Vis Absorption Spectra

**Figure 2:**
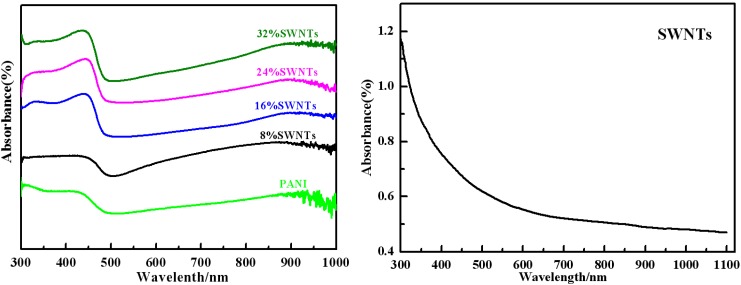
Ultraviolet-visible (UV-Vis) spectra of PANI, SWNTs and PANI/SWNTs composites with content of SWNTs varying from 8 wt% to 32 wt%.

Figure 2 shows the UV–Vis absorption spectra of PANI, SWNTs and PANI/ SWNTs composites in *m*-cresol solution. The PANI/SWNTs composites and PANI show three characteristic absorption peaks at ~315–332 nm, ~430–445 nm and 880–904 nm, which is similar to the PANI/MWNTs [9,16,17,18]. The SWNTs do not show any peaks at wavelengths ranging from 300 nm to 1100 nm. When comparing the absorption spectra, one can see that the peak positions and intensities of the composites are different from pure PANI, implying some interactions between PANI and SWNTs [19]. And the intensity ratio (A_836–863_/A_316–336_) of the composites is higher than with PANI, suggesting a higher doping level of the composites than that of PANI.

### 2.3. X-Ray Diffraction Patterns

**Figure 3:**
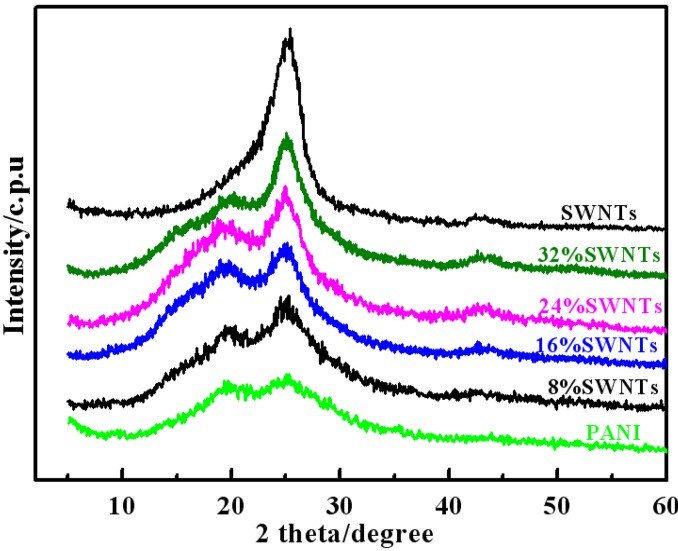
X-ray diffraction (XRD) patterns of SWNTs, PANI and PANI/SWNTs composites with a content of SWNTs varying from 8 wt% to 32 wt%.

Figure 3 shows the X-ray diffraction (XRD) patterns of PANI/ SWNTs composites, PANI and SWNTs. As shown in this Figure, SWNTs exhibit a sharp peak with high intensity at 2*θ* = ~25° and a lower intensity peak at 2*θ* = ~43°, which are in agreement with previous reports [20]. The PANI shows peaks at 2*θ* = ~20° and 25°, which are ascribed to the periodicity parallel and perpendicular to the polymer chains, respectively, and these peaks are very similar to those of chemically synthesized *p*-toluenesulphonic acid (*p*-TSA) doped PANI [21]. For PANI/SWNTs composites, the X-ray patterns show both the characteristic peaks of PANI and SWNTs, indicating the presence of SWNTs in the polymer matrix. Compared with PANI, in the patterns of composites the intensity of the peak at 2*θ* = 26° is gradually strengthened with the increase of SWNTs/aniline mass ratio, which indicates that the peak of composites at 2*θ* = 26° should be mainly owed to the overlapping of PANI and SWNTs. 

### 2.4. Morphology

**Figure 4:**
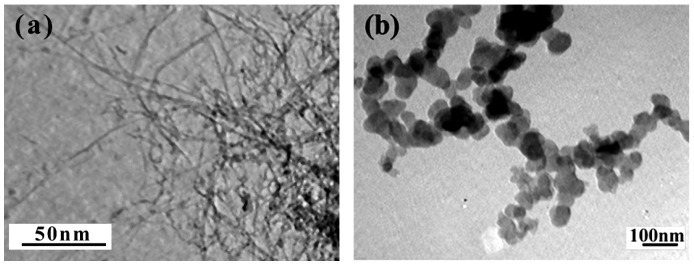
(**a**) Transmission electron microscopy (TEM) images of the SWNTs; (**b**) TEM images of PANI.

Figure 4 shows the TEM images of SWNTs and PANI, respectively. In Figure 4(a), the SWNTs are aggregated, the wall and central hollow tubular structure cannot be seen clearly, which results from the smaller size (outer diameter <2 nm), higher entanglement and closer packing tendency of SWNTs than MWNTs. In comparison, the PANI particles display granular-like particles with a size smaller than 100 nm, as shown Figure 4(b). 

**Figure 5:**
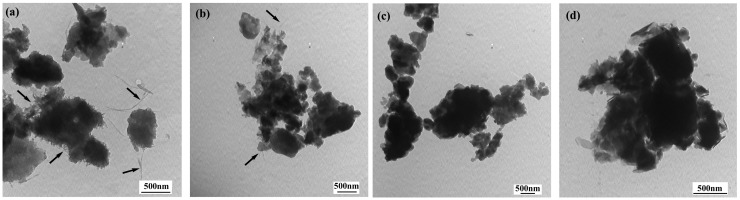
TEM images of the PANI/SWNTs composites with SWNTs: (**a**) 8 wt%; (**b**) 16 wt%; (**c**) 24 wt%; (**d**) 32 wt%.

Figure 5 shows the TEM images of PANI/SWNTs composites. The TEM images of the samples are found to vary with the content of SWNTs. It is clear from these images that the presence of SWNTs in the reaction system highly affects the morphology of PANI. Furthermore, the size and shape of composite particles are irregular. These morphologies are quite different from the PANI/carbone nanotubes composites synthesized by chemical or in-situ oxidative polymerization methods [4,22]. Comparing the TEM images of the composites, it can be found that the enwrapped SWNTs are visible in the case of composites with 8 wt% SWNT, but it becomes more difficult to observe any enwrapped SWNTs in the case of other composites. In our previous report, we observed the rod-like and granular-like features for PANI/MWNTs, which are supposed to be the PANI occurring at the inter-tube contacts [9]. The strong differences in morphology of PANI/SWNTs and PANI/MWNTs can be attributed to the higher entanglement and closer packing tendency of SWNTs than MWNTs, which prohibits the uniform dispersion of SWNTs in a polymer matrix. Therefore, the visible enwrapped SWNTs in the case of composites with 8 wt% SWNTs can result from lower amounts of SWNTs (8 wt%), which can facilitate the dispersion of SWNT in a polymer matrix. 

**Figure 6:**
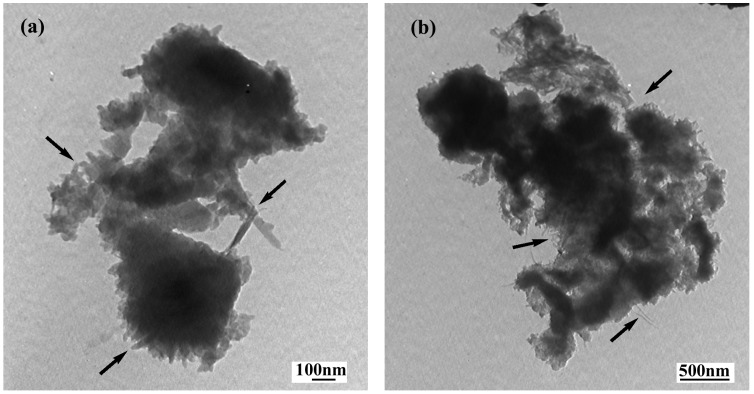
TEM images of the PANI/SWNTs composites with 8 wt% SWNTs from different scopes and magnifications: (**a**) 60,000×; (**b**) 25,000×.

Further investigation of the morphology of composite with 8 wt% SWNT is shown in Figure 6, which gives the TEM images from different scopes and magnifications. One can easily see the enwrapped SWNTs in these TEM images. Furthermore, the pore structural characteristic of this composite is obvious from these TEM images. 

### 2.5. Galvanostatic Charge-Discharge Curves

**Figure 7:**
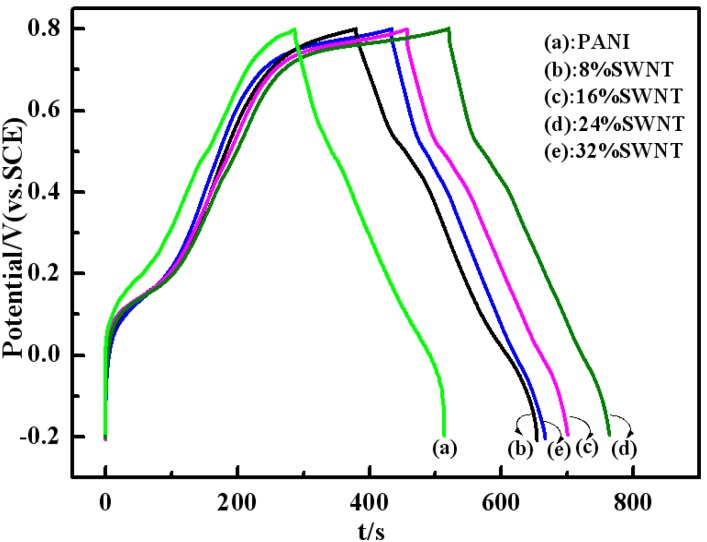
Galvanostatic charge-discharge curves of PANI and PANI/SWNTs composites with a content of SWNTs varying from 8 wt% to 32 wt% at current density of 5 mAcm^−2^ in 1 M H_2_SO_4_. Mass of the active material: 3 mg.

Figure 7 shows the galvanostatic charge-discharge curves of PANI and PANI/SWNTs composites with a content of SWNTs varying from 8 wt% to 32 wt% at a current density of 5 mAcm^−2^ in a three-electrode system between −0.2 and 0.8 V. The supporting electrolyte is 1 M H_2_SO_4_. The specific capacitance (SC) of the electrode material is calculated by means of SC = (*I* × Δ*t*)/(Δ*V* × m) [23], where *I* is the charge-discharge current, Δt is the discharge time, Δ*V* is the electrochemical window (1 V), and *m* the mass of active materials within the electrode (3 mg). The specific capacitances of PANI and PANI/SWNTs composites calculated from Figure 7 are 370 (PANI), 423 (8 wt% SWNTs), 408 (16 wt% SWNTs), 405 (24 wt% SWNTs) and 388 (32 wt% SWNTs) Fg^−1^ at 5 mAcm^−2^, respectively. In comparison with the pure PANI, the composites show a higher SC. This enhanced SC may result from the contribution of SWNTs, which can improve the capacitance of the PANI with their good conductivity. Furthermore, the presence of surface functionalized SWNTs may increase the surface area of the composites and allow excellent electrolyte access in three dimensions [24]. It should be noted here that the highest SC of PANI/SWNTs is 423 Fg^−1^ (8 wt% SWNTs), which is lower than that of PANI/MWNTs (515 Fg^−1^, 16 wt% MWNTs). A possible reason for such a difference between the SC values of PANI/SWNTs and PANI/MWNTs may be related to the higher SC of acid treated MWNTs than SWNTs [25]. 

**Figure 8:**
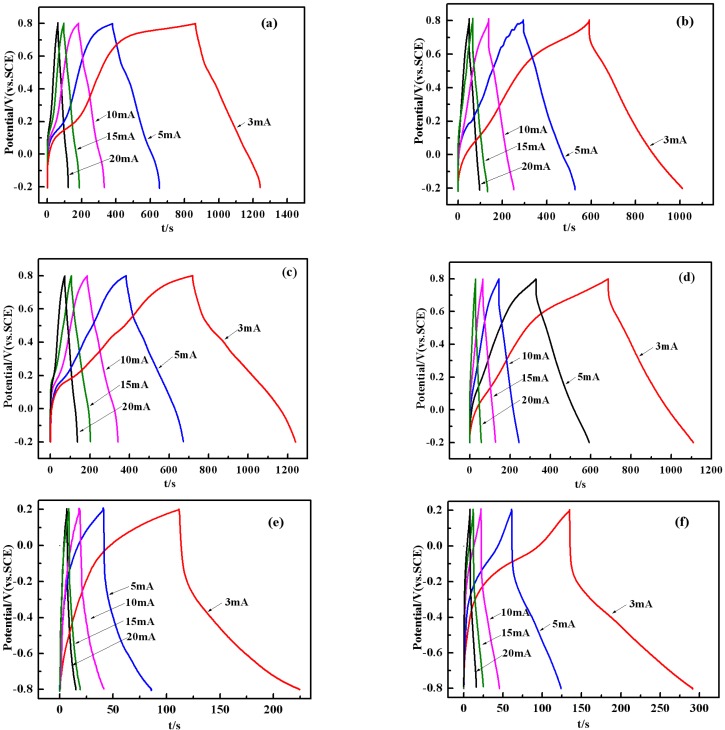
Galvanostatic charge-discharge curves of 8 wt% SWNTs composite: (**a**) in 1M H_2_SO_4_ and (**b**) in 1 M KCl. Galvanostatic charge-discharge curves of 16 wt% MWNTs composite: **(c**) in 1 M H_2_SO_4_ and **(d)** in 1 M KCl. Galvanostatic charge-discharge curves of 8 wt% SWNTs composite in 6 M KOH (**e**); Galvanostatic charge-discharge curves of 16 wt% MWNTs composite in 6 M KOH (**f**).

The further galvanostatic charge–discharge curves and capacitance retention of PANI/SWNTs composites with 8 wt% SWNTs in 1 M H_2_SO_4_, 1 M KCl and 6 M KOH at different current densities of 3, 5, 10, 15 and 20 mAcm^−2^ are given in Figure 8. For comparison, the galvanostatic charge–discharge curves of 16%MWNTs composite are also given in Figure 8. It can be seen from Figure 8(a,b) that all the curves of 8 wt% SWNTs both in 1 M H_2_SO_4_ and 1 M KCl are not ideal straight lines, indicating the process of a faradic reaction. The SC of the composites calculated from Figure 8(a) are 378, 423, 501, 451 and 396 Fg^−1^, respectively, while the corresponding values from Figure 8(b) are 418, 389, 377, 328 and 313 Fg^−1^, respectively.

In comparison to the 8 wt% SWNTs composite, the SC of the 16 wt% MWNTs composite calculated from Figure 8(c) are 522, 515, 495, 485 and 430 Fg^−1^ at the current density of 3, 5, 10, 15 and 20 mAcm^−2^, respectively, while the corresponding values from Figure 8(d) are 425, 415, 335, 310 and 187 Fg^−1^, respectively. Figure 8(e,f) shows the galvanostatic charge–discharge curves of 8 wt% SWNTs and 16 wt% MWNTs composite in 6 M KOH, and the SC of the composite calculated from Figure 8(e) for 8 wt% SWNTs in 6 M KOH are 114, 77, 76, 53 and 17 Fg^−1^, while the corresponding values from Figure 8(f) for 16 wt% MWNTs are 157, 105, 80, 65 and 53 Fg^−1^. It should be noted that the SC of the composites are quite small in the case of 6M KOH (Figure 8(e,f)), indicting poor conductivity of these composites in alkaline solution at this negative potential range, which is consistent with previous reports [26]. Furthermore, it can be concluded that the 8 wt% SWNTs composite shows a lower SC than 16 wt% MWNTs in different electrolytes.

**Figure 9:**
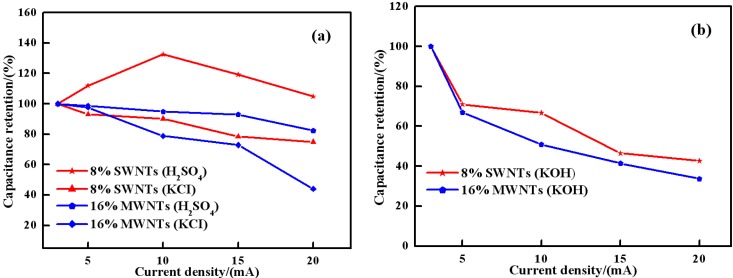
(**a**) Capacitance retention of composites in 1 M H_2_SO_4_ and in 1 M KCl; (**b**) Capacitance retention of composites in 6 M KOH.

The variations in the capacity retention as a function of the current density of 8 wt% SWNTs and 16 wt% MWNTs composite in 1 M H_2_SO_4_, 1 M KCl and 6 M KOH are plotted in the Figure 9. It can be seen from Figure 9(a) that the capacity retention ratio of 8 wt% SWNTs composite in 1M H_2_SO_4_ and 1 M KCl is 105% and 75%, respectively, when the current density varies from 3 mAcm^−2^ to 20 mAcm^−2^, while the corresponding values of 16% MWNTs are 83% and 44%. In the case of 6 M KOH (Figure 9(b)), the capacity retention of both composites decreases greatly with increase of current density. However, the 8 wt% SWNTs composite displays a higher capacity retention in 6M KOH than that of 16% MWNTs composite. With regard to electrical conductivity, metallic SWNT rope exhibits values around 10,000–30,000 S/cm [27], while electrical conductivities of approximately 200–2000 S/cm have been obtained for individual MWNTs [28,29]. Additionally, SWNTs possess a higher surface area, interconnectivity as well as mechanical strength than MWNTs [30]. These features may allow excellent electrolyte access and prevent the electrochemical degradation of PANI during the redox processes. Therefore, SWNTs composites exhibited more stable electrochemical behavior than MWNTs composites in acidic, neutral and alkaline electrolyte.

**Figure 10:**
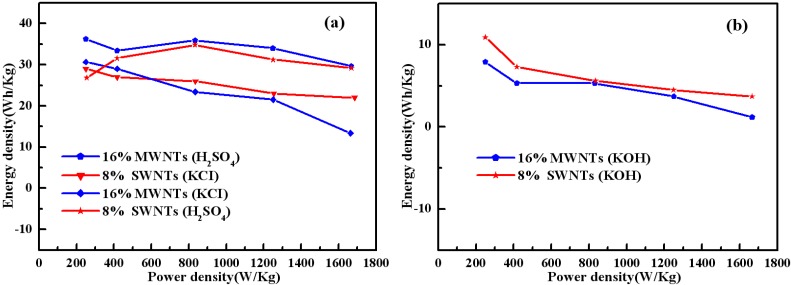
(**a**) Regone plots of specific power *vs*. specific energy for composites in 1 M H_2_SO_4_ and in 1 M KCl; (**b**) Regone plots of specific power *vs*. specific energy for composites in 6 M KOH.

Figure 10 compares the specific energy density (E) and power density of 8 wt% SWNTs and 16 wt% MWNTs composite in 1 M H_2_SO_4_, 1 M KCl and 6 M KOH. The specific energy density (E) and power density (P) are evaluated according to Equations E = (C_m_V^2^)/4 and P = E/t [31], with C_m_ being the specific capacitance of the composite electrode, t the discharge time and V = 1 V. As shown in Figure 10(a), the specific energy densities of two kinds of composites get the highest value at the power density of 833 W/kg in 1 M H_2_SO_4_, then decrease with increasing power density. In the case of 1 M KCl and 6 M KOH, the specific energy densities of two kinds of composites reduce with increasing power densities. The energy density of the composites in 1 M H_2_SO_4_ can reach 36 W h/kg at a power density of 833 W/kg, and still remains 29 W h/kg at a power density of 1665 W/kg. In comparison, Zhang *et al*. [31] studied PANI/ MWNTs prepared by microwave-assisted method, and the electrochemical results showed that the specific energy density of PANI/ MWNTs reduced slowly with increasing power densities. Furthermore, the energy density of the composite can reach 22 W h/kg at a power density of 83 W/kg, and still remains 19.4 W h/kg at a power density of 415 W/kg, which is lower than that of the composites in this case. Thus, the solid-state synthesis method is proved to be an effective strategy in preparing PANI/carbon nanotube composites with good electrochemical performance.

**Figure 11:**
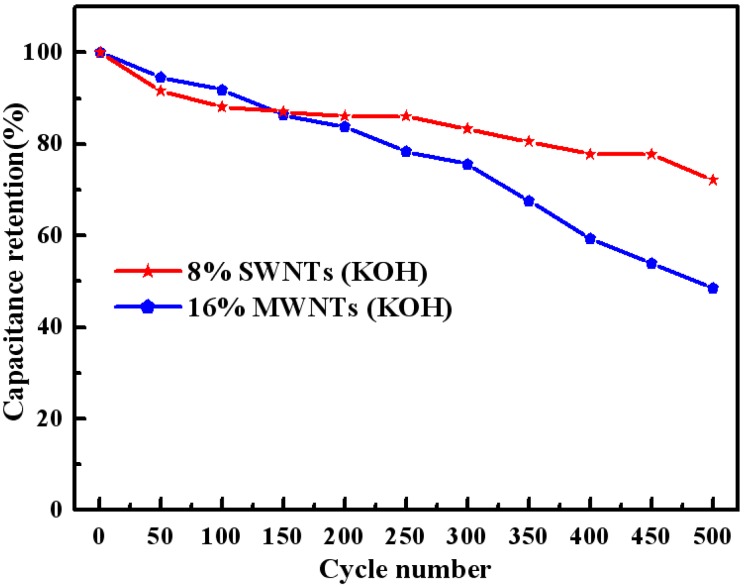
Variation of specific capacitance with cycle number for the composites in 6 M KOH at a current density of 3 mAcm^−2^.

In order to further understand the cycle life of this composite, the long-term cycling stability of the composites was also evaluated by repeated galvanostatic charge–discharge cycling (over 500 cycles) in a two-electrode system consisting of two identical working electrodes in 6 M KOH. The SC of the composites in the two-electrode system are presented in Figure 11. As can be seen from Figure 11, in comparison with the 8 wt% SWNTs composite, the rapid loss in SC is clearly found in the case of the 16 wt% MWNTs composite. This result further confirms that the SWNTs composites exhibit more stable electrochemical behavior than MWNTs composites in alkaline electrolyte.

## 3. Experimental Section

### 3.1. Materials

Aniline and ammonium peroxydisulfate ((NH_4_)_2_S_2_O_8_, APS) of analytical-reagent grade were obtained from Xi’an Chemical Reagent Company (China), *p*-toluenesulphonic acid (*p*-TSA) was obtained from Acros Organics. Single-walled carbon nanotubes (SWNTs, outer diameter: <2 nm; length: 5–15 μm) and multi-walled carbon nanotubes (MWNTs, outer diameter: <10 nm; length: 5–15 μm) were obtained from Shenzhen Nanoport Corporation (China), and they were purified by refluxing in 6 M HNO_3_ for 5 h, and then thoroughly washed with deionized water and ethanol before being dried at 60 °C for 24 h. Aniline was purified by distillation under reduced pressure. All other chemicals and solvents were used as received without further purification.

### 3.2. Preparation of PANI/SWNTs Composites

The typical solid-state synthesis procedure was as follows: Aniline (5 mmol) was added slowly to the vessel containing purified SWNTs (0.04 g), and the mixture was ultrasonicated for 30 min to facilitate the adsorption of aniline onto the wall of SWNTs. After ultrasonication, they became sponge like mixtures, then *p*-TSA (0.95 g) was added to the mixture to form a grey powder. The grey powder was put into the mortar for grinding for about 10 min, then 1.1 g of APS was added and the mixture was further ground for 40 min until the color of the solid changed to greenish black. The greenish black powder was washed with ethanol and distilled water until the filtrate was colorless, then the powder was dried under vacuum at 60 °C for 48 h. Several PANI/SWNTs composites were synthesized with different wt% of SWNTs with respect to the aniline. For comparative purposes, pristine PANI was synthesized under similar conditions without using SWNTs. The yields of samples were 77%, 71%, 70% and 80% for the composites, respectively, and 68% for PANI.

### 3.3. Structure Characterization

The fourier transform infrared (FTIR) spectra of the composites were obtained by using a BRUKERQEUINOX-55 fourier transform infrared spectrometer (Billerica, MA) (frequency range 4000–500 cm^−1^). UV–vis spectra of the samples were recorded on a Shimadzu UV-2450 spectrophotometer. The X-ray diffraction (XRD) studies were performed on a D/Max 2400 X-ray diffractometer by using CuKα radiation source (*λ* = 0.15418 nm), the scan range (2*θ*) was 5°–60°. Transmission electron microscopy (TEM) experiments were carried out in a Hitachi 2600 electron microscope. The samples for TEM measurements were prepared by placing a few drops of PANI/SWNTs composite ethanol suspension on copper supports.

### 3.4. Electrochemical Tests

The working electrodes were prepared by mixing 85 wt% active materials (3 mg), 10 wt% carbon black and 5 wt% polytetrafluoroethylene (PTFE) to form a slurry. The slurry was pressed onto a graphite current collector (area: 1 cm^2^), then dried at 60° for 24 h. Half-cell electrode tests were performed with the three-electrode cells using a standard calomel reference electrode (SCE) and a Pt foil of counter electrode in the electrolytes of 1 M H_2_SO_4_ and 1M KCl, while the SCE was replaced by the Hg/HgO reference electrode in the case of 6M KOH. The galvanostatic charge–discharge tests were performed in the potential window ranging from −0.2 to 0.8 V in 1 M H_2_SO_4_ and 1 M KCl, while the tential window ranged from −0.8 to 0.2 V in 6 M KOH, The cycle life measurement of composite electrode was recorded by repeated galvanostatic charge–discharge cycling (over 500 cycles) in two-electrode system consisted of two identical working electrodes. The electrolyte was 6 M KOH, and the potential window ranged from −0.8 to 0.2 V. All electrochemical performances were done using the CHI660C electrochemical working station.

## 4. Conclusions

In this work, PANI/SWNTs composites with a content of SWNTs varying from 8wt% to 32wt% were synthesized by solid-state synthesis method. The experimental results were compared with those of PANI/SWNTs and those of PANI/MWNTs prepared under the same polymerization conditions. The comparison results indicated that the effect of the SWNTs on the PANI was similar to that of PANI/MWNTs, in which the PANI displayed a higher oxidation and doping degree than pure PANI, implying the presence of some interactions between PANI and SWNTs or MWNTs. The comparison results also suggested that the higher entanglement and closer packing tendency of SWNTs than MWNTs caused a different morphology for PANI/SWNTs compared with that of PANI/MWNTs. Although the specific capacitance of PANI/MWNTs is higher than that of PANI/SWNTs, the higher conductivity, surface area, interconnectivity as well as mechanical strength than MWNTs led the PANI/SWNTs exhibiting a more stable electrochemical behavior than PANI/MWNTs both in neutral and alkaline electrolytes. All electrochemical results also suggest that the solid-state synthesized PANI/SWNTs composites can be used as electrode material for supercapacitors.
